# A Scientometric Study of PubMed-Indexed Endodontic Articles: A Comparison between Iran and Other Regional Countries

**Published:** 2012-06-01

**Authors:** Mohammad Jafar Eghbal, Negar Davari Ardakani, Saeed Asgary

**Affiliations:** 1. Dental Research Center, Iranian Center for Endodontic Research, Dental School, Shahid Beheshti University of Medical Sciences, Tehran, Iran; 2. Linguistics Department, Literature Faculty, Shahid Beheshti University of Medical Sciences, Tehran, Iran; 3. Iranian Center for Endodontic Research, Dental Research Center, Dental School, Shahid Beheshti University of Medical Sciences, Tehran, Iran

**Keywords:** Asians, Biomedical Research, Endodontic, Iran, Publications, Pubmed, Research Activities, Scientometric

## Abstract

**Introduction:**

Based on Iran’s 20-year prospective national vision document, Iran may be seen as the most developed country in science and technology in the region by 2025. In this report, bibliometric indicators are used to compare the research productivity in endodontics between Iran and 28 selected Asian countries.

**Materials and Methods:**

Medical Subject Headings keyword-searching of PubMed up to 2012 was conducted to compare the scientific publications in Iran and neighboring countries (Middle East, Middle Asia, Caucasus and EMRO countries). Highest 5 PubMed-indexed endodontic journals were also searched to retrieve the number of published articles of the top five countries. Data were extracted, tabulated and compared to identify the ranks as well as trends.

**Results:**

The results confirm that there are many differences in scientific endodontic publications between the studied countries; Iran ranked second in endodontic research productivity compared to Turkey in 2011. However, Iran attained first place in 2010 as well as the most positive publication trend in PubMed-indexed endodontic journals.

**Conclusion:**

Although Iran was in second rank in the region last year, the positive trend in published endodontic research papers is considered a sign of future success in acquiring Iran’s vision.

## Introduction

Scientometrics is the science of measuring and analyzing science using qualitative, quantitative and computational approaches. Scientometrics with its various indices is a reliable method for evaluation of scientific development [1]. One of its main indices is the number of published articles or science production in a specific field of science. Based on Iran’s 20-year national vision, Iran wishes to gain the first economic, scientific, and technologic rank amongst the South West Asia region countries.

During 1979-2009 the number of Persian/English medical journals increased from 8 to 146 [2]. According to Aminpoor et al.’s report in 2009, among the 141 Iranian Persian/English medical journals, only 16 were documented in ISI, while none of the dental journals were published in PubMed [3]. A survey on science production during the past decades indicates a rapid growth in the field of dentistry. Iran’s rank in the year 1999 to 2003 was 43rd in the world and 14th amongst Asian countries. During the period from 1979 to 2006, 2292 Persian articles and 104 English articles were published from Iranian scholars, none of which were documented in the Web of Science [2].

The first Iranian dental journal was published in English in 2004 and the number of English journals increased to 7 by 2011; two have been indexed in PubMed [3]. Serati Shirazi et al. (2011) reported 5.68% growth in science production in dentistry during 2000-2009, according to Web of Science database [4]. It has been reported that less than 7% of Iran's medical articles were published in international reputable databases [3].

A recent report analyzed endodontic articles that were published in PubMed-indexed journals, Medical Universities as well as dentists with the greatest number of articles were identified [5]. The aim of this report was to compare Iran’s published endodontic articles in PubMed-indexed journals with South West Asia region countries including Middle East, Middle Asia and Caucasus as well as WHO Eastern Mediterranean Region (EMRO) countries.

## Materials and methods

A search strategy based on Medical Subject Headings keyword-searching for endodontic subjects was implemented (Table 1).The MESH words were utilized for identifying PubMed-indexed endodontic articles.

**Table 1:** Endodontic terms included in Medical Subject Headings (MeSH)

endodontic	dental pulp test	dental pulp diseases
endodontics	root canal irrigants	pulpitis
root canal therapy	root canal filling materials	dental pulp exposure
root canal preparation	rubber dams	periapical abscess
dental pulp capping	gutta-percha	dental pulp calcification
pulpotomy	retrograde obturation	dental pulp devitalization
pulpectomy	mineral trioxide aggregate	periapical periodontitis
apicoectomy	dental pulp cavity	dental pulp necrosis
tooth replantation	smear layer	pulp capping agentsd
dental implantation	endosseous implantation	pulpectomy agents

The name of neighboring, Middle East, Middle Asia, Caucasus and EMRO countries were identified according to free encyclopedia of Wikipedia. After omission of overlapping names, the final list included the following 29 countries: Afghanistan, Algeria, Armenia, Azerbaijan, Bahrain, Djibouti, Egypt, Georgia, Iran, Iraq, Israel, Jordan, Kuwait, Lebanon, Libya, Morocco, Oman, Pakistan, Palestine, Qatar, Russia, Saudi Arabia, Somalia, Sudan, Syria, Tunisia, Turkey, United Arab Emirates, and Yemen.

Subsequently, the PubMed-indexed endodontic journal articles were searched with the name of each country without time limitation to recognize the total publications of each country. The top five countries were then searched with time limitation to identify the trends.

“Journal of Endodontics”, “International Endodontic Journal”, “Oral Surgery, Oral Medicine, Oral Pathology, Oral Radiology and Endodontology”, “Endodontics and Dental Traumatology”, and “Australian Endodontic Journal” were searched to retrieve the total number of PubMed-indexed endodontic articles of the top five countries.

## Results

Before 2012, there were 41,301 endodontic articles published on endodontics and cited in PubMed. The number of endodontic articles in 29 countries is listed in Table 2.Turkey stands as number one with the greatest total number of articles (962), followed by Israel, Iran, Jordan, and Saudi Arabia. Again, Turkey achieves number one status in 2011 with its 81 published endodontics articles. Iran achieved second rank by publishing 54 articles; followed by a large margin by Israel, Saudi Arabia, Egypt and Jordan are next in line.

**Table 2:** The number of articles in 2011 and the total number of PubMed-indexed endodontic articles for the 29 studied countries

**Country**	**2011**	**Total **
Turkey	81	962
Iran	54	322
Saudi Arabia	14	109
Israel	13	385
Egypt	13	102
Jordan	6	66
Kuwait	3	31
Iraq	2	12
Tunisia	2	5
Morocco	2	3
Lebanon	1	32
Sudan	1	10
Bahrain	1	3
Russia	0	24
Pakistan	0	9
United Arab Emirates	0	4
Oman	0	4
Georgia	0	3
Syria	0	3
Libya	0	2
Armenia	0	1
Qatar	0	0
Yemen	0	0
Palestine	0	0
Algeria	0	0
Azerbaijan	0	0
Afghanistan	0	0
Djibouti	0	0
Somalia	0	0

Total number of published articles of the 5 most related journals was retrieved and demonstrated in first column of Table 3. The total number of PubMed-indexed articles as well as the number of published articles in 2011 for top 5 countries were quantified and tabulated (Table 3).

**Table 3:** Total number of PubMed-indexed endodontic articles of top five countries (and published articles in 2011) in 5 highest ranked endodontic journals

**Journal ***(Total number of articles)*****	**Turkey**	**Iran**	**Saudi ** **Arabia**	**Israel**	**Egypt**
*Oral Surgery, Oral Medicine, Oral Pathology, Oral Radiology and Endodontics (5499)*	292 (26)	42 (4)	25 (3)	121 (7)	14 (4)
*Journal of Endodontics (5483)*	178 (12)	55 (9)	17 (2)	84 (2)	19 (5)
*International Endodontic Journal (2255)*	108 (14)	30 (8)	10 (1)	25 (1)	3 (1)
*Endodontics and Dental Traumatology (989)*	132 (12)	10 (6)	5 (2)	54 (5)	0 (0)
*Australian Endodontic Journal (346)*	10 (6)	34 (0)	2 (0)	0 (0)	1 (0)

**Figure 1:**
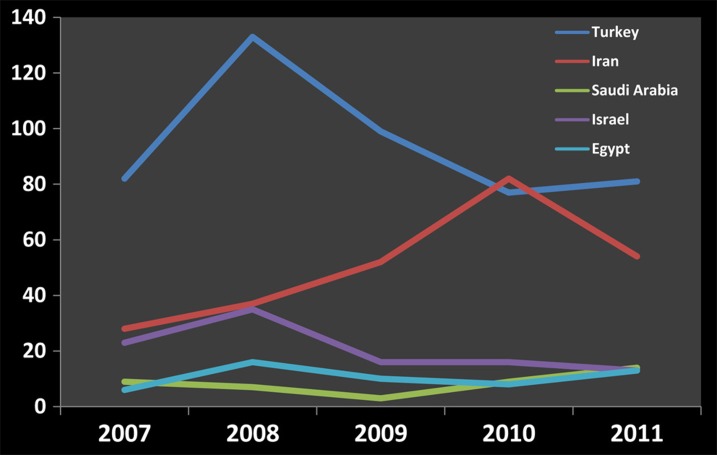
The trend of published PubMed-indexed endodontic articles of the top five countries

The number of PubMed-indexed endodontic articles of the top five countries was counted and analyzed over this 5 year period (2007-2011) to identify the trends of scientific publication (illustrated in Figure 1).

## Discussion

On the basis of Iran’s 20-year national vision document, Iran is pictured as the highest developed country in science/technology by 2025. As measured by the Web of Science, the rate of science production (GI; growth index) from 1980-1994 to 1995-2009 at the country level had the fastest growth in Iran (GI of 14.4) followed by Republic of Korea (GI=9.8), Turkey (GI=7.8), Cyprus (GI=5.2), China (GI=5.1), and Oman (GI=4.8); the average growth in the world is 1 (GI). Israel, Saudi Arabia and Iraq have grown at a slower rate than the world level. In Africa, growth was strongest in Tunisia (GI=3.2), Morocco (GI=2.8) and Algeria (GI=2.7). Growth index for the most developed countries is at a slower pace compared with the world rate i.e. USA (GI=0.77), Canada (GI=0.82) and UK (GI=0.86) [6]. During the past two decades, Iran has demonstrated one of the fastest growth rates in scientific production in the whole world [6]. The results of the present study verify Iran's aforementioned growth rate.

Iran has experienced a considerable growth in PubMed-indexed endodontic articles [5] with a total documented 82 and 54 endodontics articles in 2010 and 2011, respectively. In 2010,though Iran was amongst the top countries it stood in 2011 behind Turkey with a significant difference. Therefore, implementing a compensating policy is highly recommended.

The increase in the number of dental faculties as well as research centers and consequently the increase of endodontic specialists, students and also research projects and dissertations are inevitably positive factors which influence the rise in the number of articles in this field.

The dominant scientific publication language is English as a result a general trend towards English journals has been observed. Therefore, competency in English could also be considered an influential factor in the increase of PubMed-indexed articles in English journals. Focus on gaining competency in English has considerably increased in recent years and this may lead into a great and outstanding growth rate of articles in the following years.

## Conclusions

A positive trend exists in Iran for PubMed-indexed endodontic articles; however, research policy makers in endodontics should find solutions to improve Iran’s science system quality to further this growth. Moreover, focus should be on the vision behind the research; that is, the quality of the research and its influence on improving endodontology.
